# The Harmonization of Four Delirium Instruments

**DOI:** 10.1093/geroni/igaa057.1678

**Published:** 2020-12-16

**Authors:** Benjamin Helfand, Elke Detroyer, Koen Milisen, Dimitrios Adamis, Richard Jones

**Affiliations:** 1 University of Massachusetts Medical School, Newton, Massachusetts, United States; 2 KU Leuven, Leuven, Netherlands; 3 KU Leuven, LEUVEN, Vlaams-Brabant, Belgium; 4 Sligo Mental Health Services, Sligo, Ireland; 5 Brown University, Providence, Rhode Island, United States

## Abstract

Delirium is a clinical syndrome characterized by acute cognitive dysfunction, which is pervasive in older persons. Delirium affects over 2.6 million Americans over age 65 annually. One major problem in detection of delirium is that over 40 different instruments have been created to identify delirium in different clinical settings. There is no single agreed upon reference standard instrument. In previous work, we performed a systematic review to identify the four most commonly cited and well-validated instruments for delirium identification. The aim of this study is to harmonize these four commonly used instruments: Confusion Assessment Method (CAM), Delirium Observation Screening Scale (DOSS), Delirium Rating Scale-Revised-98 (DRS-R-98), and Memorial Delirium Assessment Scale (MDAS). We used data from three separate sources (N = 1623). Participants were rated by multiple and overlapping instruments across studies, allowing us to apply item response theory linking procedures. We fit generalized structural equation models, and found unidimensional factor models fit well. We found the instruments were highly correlated (all r > 0.9) and kappa statistics for delirium case identification were high (range: 0.89 to 0.95). We generated crosswalks to map sum scores on one instrument to another. Our results suggest the same underlying construct, propensity to delirium, is measured across the four instruments. The crosswalks facilitate comparison and combination for immediate clinical use or for future meta-analyses. In future steps, we will use our results to find the optimal cut-points for use across all instruments to identify delirium.

